# Discrimination of Motion Direction in a Robot Using a Phenomenological Model of Synaptic Plasticity

**DOI:** 10.1155/2019/6989128

**Published:** 2019-05-02

**Authors:** Nareg Berberian, Matt Ross, Sylvain Chartier

**Affiliations:** Laboratory for Computational Neurodynamics and Cognition, School of Psychology, University of Ottawa, Ottawa, ON, Canada K1N 6N5

## Abstract

Recognizing and tracking the direction of moving stimuli is crucial to the control of much animal behaviour. In this study, we examine whether a bio-inspired model of synaptic plasticity implemented in a robotic agent may allow the discrimination of motion direction of real-world stimuli. Starting with a well-established model of short-term synaptic plasticity (STP), we develop a microcircuit motif of spiking neurons capable of exhibiting preferential and nonpreferential responses to changes in the direction of an orientation stimulus in motion. While the robotic agent processes sensory inputs, the STP mechanism introduces direction-dependent changes in the synaptic connections of the microcircuit, resulting in a population of units that exhibit a typical cortical response property observed in primary visual cortex (V1), namely, direction selectivity. Visually evoked responses from the model are then compared to those observed in multielectrode recordings from V1 in anesthetized macaque monkeys, while sinusoidal gratings are displayed on a screen. Overall, the model highlights the role of STP as a complementary mechanism in explaining the direction selectivity and applies these insights in a physical robot as a method for validating important response characteristics observed in experimental data from V1, namely, direction selectivity.

## 1. Introduction

Although a seemingly effortless task for humans, recognizing and tracking the direction of visual objects is based on an incredible complexity of brain areas involved in visual processing and attention, as well as learning and memory. In recent years, the advent of artificial neural networks (ANNs) has allowed the combination and isolation of the interactions of important biophysical mechanisms in order to shed light on the nature of biological phenomena. Through a symbiotic collaboration between neuroscience and artificial intelligence, the application of ANNs is in part to unify our understanding of the underlying mechanisms contributing to sensory experience. The development of these brain-inspired computational systems have shown their usefulness in revealing novel mechanisms of neuronal circuitry and in proposing experimental predictions that can be directly tested in experimental settings. In order to elucidate the circuit mechanisms underlying visual perception, mathematical models have been formulated with strong support from electrophysiological data [1]. Due to their usefulness and their predictive ability in driving new neuroscientific discoveries, brain-inspired ANNs also have the potential to be implemented in robotic agents in order to further assess their ecological validity [2]. Given that mechanistic models cannot yet capture the full complexity of the nature of perceptual phenomena, the implementation of well-established models from neuroscience into the domain of artificial intelligence opens new avenues for understanding biological networks exposed to real-world stimuli [3]. Previous approaches in modelling the perceptual phenomena of motion have shown successful attempts in incorporating natural visual inputs in networks of spiking neurons [4–6].

In this study, we propose a model of motion discrimination using a ubiquitous mechanism in neuronal circuits, namely, short-term plasticity (STP), whereby the strength of synaptic connections varies from milliseconds to seconds as a result of recent activity [7, 8]. These rapid changes in synaptic strength vary overtime from one spike to the next due to short-term facilitation (STF) and short-term depression (STD) [9]. Short-term synaptic plasticity serves diverse functions in bio-inspired networks. For example, STP can process temporal patterns [10], modify a neuron's sensitivity to the temporal coherence of inputs [11, 12], participate in gain control [12], reduce redundancy [13], act as an adaptive filter [14], and improve discriminability [15] among others [16]. Despite the beneficial effects of STP on cortical computation [7, 8, 10, 16–18], it remains unclear whether STP contributes independently of sensory experience or whether it provides a causal contribution to experience-dependent plasticity. A study in-line with the former found that alteration in STP has been observed in cultural neurons, suggesting that endogenous neuronal activity (i.e.,independent of sensory experience) is sufficient to drive changes in STP [19]. In contrast, there is evidence to suggest that STP is a consequence of experience-dependent plasticity in local neuronal circuits and therefore causally linked to visually driven inputs [20–25]. For example, sensory deprivation can alter STP, but in most cases, the dynamics of synaptic transmission are often inconsistent in these experiments, as even at the same synapse type, some promote facilitation while others will exhibit depression. Nonetheless, evoked and spontaneous vesicle release is likely to be controlled by two independent and nonoverlapping mechanisms [26]. Sensory experience can therefore modify the dynamics of STP, thus pointing towards a causal contribution of STP to experience-dependent plasticity. Indeed, an important determinant of development and sensory-driven alteration in STP is the expression of presynaptic NMDA receptors (preNMDARs) [27, 28]. These are ligand-gated ionotropic glutamate receptors that serve diverse functions ranging from the coincidence detection in Hebbian learning to excitatory neurotransmission critical for information processing in the mammalian central nervous system [29].

In layers 2/3 (L2/3) of the primary visual cortex (V1), individual neurons respond more strongly to an object (i.e.,orientation grating) moving in a particular direction (“preferred”) than the same object moving in the opposite direction (“null”); a visual response property termed direction selectivity. There is surmounting experimental and theoretical evidence that STP contributes to the enhancement of motion discrimination [30–33]. In-line with previous studies, we recently found that rapid changes in synaptic strength via STP may provide an essential contribution for accurate motion discrimination [34]. Starting with the well-established Tsodyks–Markram model [1], we implement STP in the synaptic connections of a microcircuit motif. We then examine neuronal responses to changes in the direction of real-world vertical orientation stimuli moving in bidirectional motion along a single axis of motion. Furthermore, we compare neuronal responses in real-time from a robotic implementation to those of a simulated version of the model whereby units are instead exposed to a hypothetical version of real-world stimuli in motion. Finally, we analyse neuronal responses in V1 to drifting sinusoidal gratings and compare cortical responses to those observed in the robotic implementation. The remainder of the paper is divided as follows: Section 2 describes the architecture of the microcircuit motif, the setup of the robotic implementation, and the phenomenological model of STP and summarizes the experimental data analysis approach.  Section 3 illustrates all findings. In Sections 4 and 5, we summarize the overall insights of our work, propose future avenues, and highlight the contribution of our work to neurorobotics research.

## 2. Materials and Methods

### 2.1. Architecture

**Figure 1:**
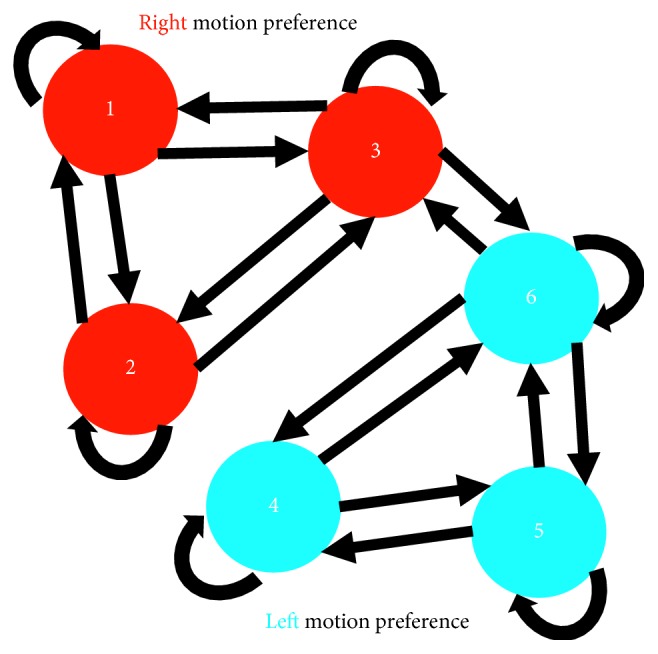
Architecture of the microcircuit. There are 6 units in total. Subpopulation 1 (red) forms a cluster of 3 units, each of which exhibits a preference for stimuli moving towards the right. The remaining units in subpopulation 2 (blue) display higher responses for stimuli moving towards the left. Connections are bidirectional, with self-connections allowed. Outgoing connections from units within both subpopulations exhibit STF.

Here, we propose a microcircuit motif of six units in total, comprised of two subpopulations connected via synapses that exhibit STF (Figure 1). This novel framework differs from our previously proposed architecture of two units mediated by STD- and STF-dominated synaptic connections [34]. In our current study, we aim to provide a more parsimonious approach in highlighting the contribution of STP by using a single STP mechanism rather than two distinct STP mechanisms for showing successful motion discrimination. In addition, we highlight the structural advantages of the expanded network over the two-unit microcircuit. Finally, we display the functional advantages resulting from the topological structure of the expanded network, which happen to be absent in a two-unit microcircuit.

In order to assess whether the embodied robot is capable of displaying response characteristics similar to those observed in local microcircuits in V1, the architecture is expanded by following a constrained network topology inspired from specific features observed in local cortical microcircuits. For example, bidirectional connectivity in V1 is a by-product of neighbouring neurons sharing similar visual responses [35]. In addition, bidirectional connectivity has been suggested to evolve according to synaptic connections mediated by STF [36]. Furthermore, neurons in V1 that share similar visual features (e.g.,similar direction preference) are more highly connected and less connected to neurons showing a reduced preference for those similar visual features [35]. Similarly, in our expanded microcircuit, there are a greater number of connections amongst units exhibiting the same direction preference and less connections between units coding for an opposing direction of motion (Figure 1) [37]. More specifically, units 1, 2, and 3 within subpopulation 1 are more highly connected amongst each other and less connected to units 4, 5, and 6 within subpopulation 2. These topological features of the expanded network would be absent in a two-unit microcircuit with bidirectional connections because both units would exhibit the same number of outgoing and incoming connections, acting as a single isolated subpopulation. Consequently, we hypothesized that, in the expanded architecture, units within subpopulation 1 and subpopulation 2 would exhibit preferential responses to opposing directions of motion. In contrast, we expected that a microcircuit of two units with bidirectional connections would have a limited functional contribution by displaying preference only for a single direction of motion. Finally, it is noteworthy to mention that the study proposed here has extended the architecture to six units, as network size from this point forward would not change the desired behaviour of individual units in the model but simply increase simulation time.

### 2.2. Setup

**Figure 2:**
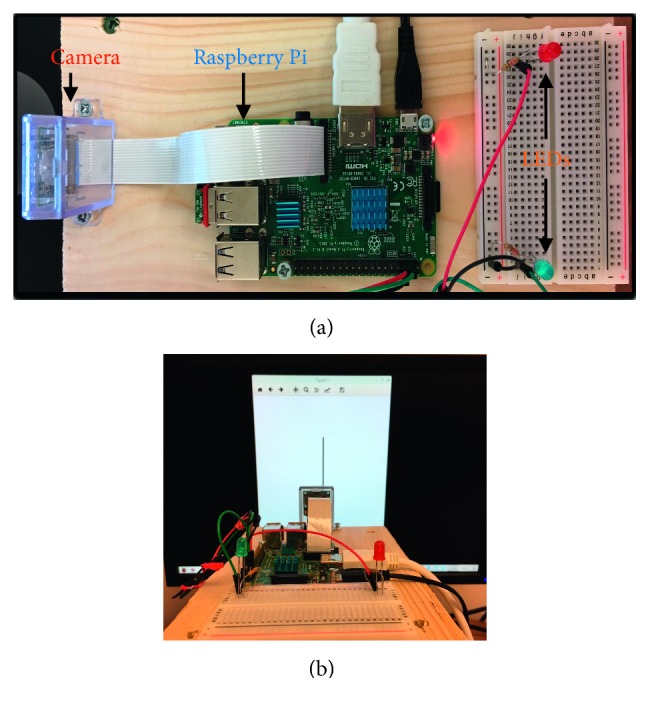
Robotic setup. (a) Setup of the camera, Raspberry Pi, and the LEDs. (b) Overview of the robotic setup in front of a computer monitor displaying an orientation stimulus from the real-world.

For the robotic implementation, we have employed the Raspberry Pi 3 Model B-V1.2 microcontroller (Figure 2(a)). To capture the image of the stimulus in motion, a Raspberry Pi camera (V2.1) is used and attached to the device via a ribbon cable. In addition, two simple circuits are created on a breadboard responsible for lighting up coloured LEDs (red and blue) and attached to the Raspberry Pi's GPIO (general purpose input/output) pins. The robotic setup is mounted onto a wooden box, and the camera is placed 12 centimeters away from the front of a computer monitor whereby real-world stimuli are displayed (Figure 2(b)).

### 2.3. Model

Using the robotic implementation, we incorporate the mechanism of STP within the microcircuit, whereby the neurotransmitter release probability in the synaptic connections evolves according to

(1) $$\begin{array}{c} \frac{du_{j}}{dt}=\frac{U-u_{j}\left(t\right)}{\tau_{\text{f}}}+U\left(1-u_{j}\left(t\right)\right)\overset{\infty }{\underset{k=0}{\sum }}\delta \left(t-t_{k}^{\left(j\right)}\right), \end{array}$$

where *δ*(*t*) is the Dirac delta function. The sum on *k* spikes is over all spike times *t*_*k*_^(*j*)^ of presynaptic neuron *j*, and *u*_*j*_(*t*) reflects presynaptic residual calcium levels. In the absence of incoming action potentials, the synapse is at a resting state with residual calcium levels *u*_*j*_(*t*_0_)=*U*. The amount of residual calcium instantaneously increases immediately after the first action potential within a spike train, and *u*_*j*_(*t*_1_)=1 − *u*_*j*_(*t*_0_) is the fraction that remains available immediately after this first event. Hence, the running variable of *U* refers to *u*_*j*_(*t*), and *U* remains a parameter that applies to the first action potential in the spike train, after which *u*_*j*_(*t*) (the running variable of *U*) decays exponentially to its resting value *U* with a time constant *τ*_f_. As a result, each time an action potential is generated, presynaptic residual calcium instantaneously rises and then recovers with a time constant *τ*_f_ between subsequent spikes. As residual calcium levels increase, a release-ready vesicle along the active zone of the presynaptic membrane terminal releases neurotransmitters onto the postsynaptic side of the synapse. During this process of exocytosis, the neurotransmitter availability within the presynaptic terminal is described according to

(2) $$\begin{array}{c} \frac{dx_{j}}{dt}=\frac{1-x_{j}\left(t\right)}{\tau_{\text{d}}}-u_{j}\left(t\right)x_{j}\left(t\right)\overset{\infty }{\underset{k=0}{\sum }}\delta \left(t-t_{k}^{\left(j\right)}\right), \end{array}$$

where *x*_*j*_(*t*) denotes the fraction of resources that remain available following vesicle release. Between subsequent spikes, *x*_*j*_(*t*) recovers back to baseline to its resting value of 1 with a time constant *τ*_d_, restoring the amount of synaptic resources available within the presynaptic terminal. The STP model allows the examination of synaptic behaviour under a relatively short timescale. Hence, here we are interested in the properties and mechanisms of plasticity over the course of milliseconds to seconds [7]. Depending on the initial setup of kinetic parameters *τ*_f_, *τ*_d_, and *U*, the STP model can mimic the effect of a depressing or a facilitating synapse (Table 1) [1]. Therefore, the mechanism of STD and STF can be distinguished using a different parameter setup in the same governing Equations (1) and (2).

**Figure 3:**
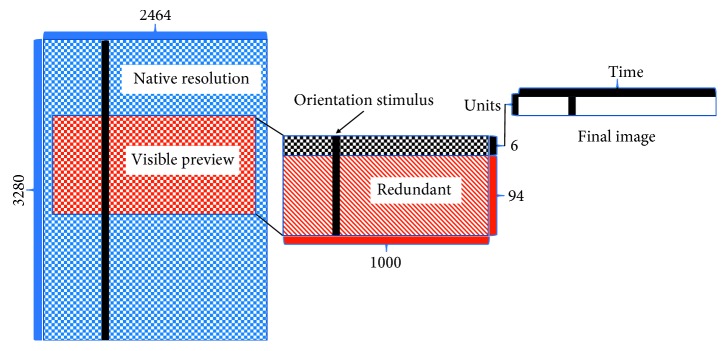
Schematic representation displaying the transformation starting from the native resolution of the Raspberry Pi camera up to the final image displayed to the robot.

In this study, images that the camera captures from the sensory environment are used as direct input into the microcircuit motif. In other words, external inputs directly generate incoming presynaptic spikes in the STP model. In order to receive synaptic inputs, the stationary camera is used to process changes in the visual image captured from the sensory environment. The generated image is displayed on a computer monitor and is characterized by a 24 bit colour scheme. This setup provides the advantage in presenting moving stimuli that follow a spatiotemporal pattern. Furthermore, we decided to mimic a similar stationary screen fixation setup used in experimental neuroscience [38–40], where an anesthetized, paralyzed animal exhibits minimal eye movements to moving stimuli displayed on the screen. As for the nature of the stimulus presented, we are interested in black and white images. Hence, an average of the three 8 bit RGB planes is taken, and the resulting plane is then converted such that every pixel inside the plane is coded by 1 (black pixel) or 0 (white pixel), instead of traditional values ranging from 0 to 255 (where 255 is the maximal intensity that could be displayed). The conversion of the image to a binary scheme allows us to directly feed the microcircuit with trains of incoming “all-or-none” action potentials as the camera processes visual images, similar to previous methods [6]. The Raspberry Pi camera has a native resolution of 3280 × 2464 (Figure 3). Two steps were applied to down sample the stimulus image and thus simplify input to the network. Firstly, the camera resolution was lowered to 100 × 1000, in order to allow a visible preview of the image processed by the camera. When the stimulus is displayed on the computer monitor, the vertical line extends across the entire first dimension and therefore activates all units in the network (Figure 3). Here, the first dimension of 100 is meant to represent the number of units to receive the input. Given that the network is comprised of six units, there are 94 remaining units along the first dimension (i.e.,rows) serving as redundant information to the network. Hence, to remove redundant information presented to the network, the dimensionality was further reduced to 6 × 1000, where each row serves as input to a single unit in the network and each column describes the amount of time needed to evaluate the activity of each unit during visual information processing.

In this way, the camera captures an image of 6000 pixels which is then directly introduced as input to the spiking network. It is noteworthy to mention that the number of presynaptic spikes that each unit receives is equal to the number of 1s encoded in the pixelated image processed from the camera. In other words, the greater the width of the orientation bar, the greater the number of pixels coded with 1s and, therefore, the higher the frequency of the presynaptic inputs. In addition, individual shifts in the orientation stimulus in motion will also shift the timing of presynaptic spikes. Finally, given the nature of the real-world stimulus, images were inherently noisy, meaning some units received a few more input spikes than others. This inherent feature potentially originated from fluctuations in luminance and/or the angle of the monitor relative to the lens of the camera.

**Figure 4:**
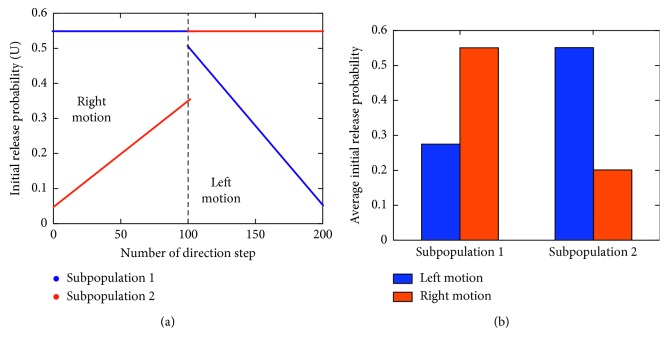
Initial release probability in the synaptic connections as a function of the direction step number. (a) Pair of initial release probabilities recruited during left and right motion discrimination. (b) Average initial release probability for left and right motion in the STF-mediated synaptic connections of subpopulation 1 and subpopulation 2.

In the model, there are a total of 200 direction steps, where each direction step represents a shift in the orientation stimulus, which is captured by using the camera. Therefore, the camera captures 200 images, 100 of which are comprised of orientation stimuli moving towards the right and the remaining 100 images are comprised of orientation stimuli moving towards the left. For individual shifts in the image, the network receives a new train of incoming presynaptic inputs for a total of 1000 ms (Table 2). When dealing with temporal coding tasks, it is necessary to manipulate the initial vesicle release probability *U* (Equation 1) [10]. Here, changes in direction steps introduce changes in the initial release probability of STF-mediated units in the microcircuit motif [34]. The range of values used to modulate the initial vesicle release probability is presented in Figure 4(a). For every direction step of the stimulus in motion, we recruited a pair of initial vesicle release probabilities, one of which was recruited by units within subpopulation 1, and the remaining one was recruited by units within subpopulation 2. Hence, a single change in the direction step introduced a new pair of initial release probabilities, which in turn mediated neuronal responses across time. Based on the temporal dynamics of synaptic transmission, units would in turn display preferential and nonpreferential responses to orientation stimuli in motion. The average initial release probability for left and right motions from each respective subpopulation shows that units within subpopulation 1 exhibit a higher initial release probability for right motion, whereas units within subpopulation 2 exhibit a higher initial release probability for left motion (Figure 4(b)).

As units receive visual input, the kinetic parameters modulate the interplay between the dynamics of *u*_*j*_(*t*) and *x*_*j*_(*t*). In turn, the joint effect of *u*_*j*_(*t*)*x*_*j*_(*t*) characterizes the short-term strength of the synaptic inputs at a given time step, thus generating an instantaneous current characterized by

(3) $$\begin{array}{c} I_{\text{i}}^{\text{stp}}\left(t\right)=A\overset{N}{\underset{j=1}{\sum }}w_{ij}u_{j}\left(t\right)x_{j}\left(t\right), \end{array}$$

where the summation is taken over all presynaptic inputs. Here, *w*_*ij*_ is the absolute synaptic efficacy from presynaptic unit *j* to postsynaptic unit *i* mediated by the temporal dynamics *u*_*j*_(*t*)*x*_*j*_(*t*) of STP [41]. For each direction step, *w*_*ij*_ is kept constant and set to 1 if unit *j* is connected to the unit *i*, otherwise it is set to 0, denoting the absence of a connection. In this way, the temporal dynamics of the instantaneous current *I*_i_^stp^(*t*) is mostly mediated by STP. *A* is a constant multiplicative factor, modulating the overall gain of the generated current. The current mediated by STP drives subthreshold membrane potential depolarization dynamics of leaky integrate-and-fire (LIF) units according to

(4) $$\begin{array}{c} \tau_{\text{m}}\frac{dV_{i}}{dt}=-V_{i}\left(t\right)+V_{\text{rest}}R_{\text{m}}I_{\text{i}}^{\text{stp}}\left(t\right), \end{array}$$

where *τ*_m_ is the membrane time constant, *I*_i_^stp^(*t*) is the current mediated by the short-term synaptic strength, and *R*_m_ is the membrane resistance. Whenever a depolarization hits a fixed threshold (*V*_*i*_(*t*) ≥ *θ*), the unit emits a spike and becomes refractory for a period *τ*_arp_, after which Equation 4 resumes from a subthreshold reset potential *V*_rest_ (Table 2).

### 2.4. Experimental Data

To draw a parallel between the responses of spiking units observed in the real-time robotic implementation versus those observed in an experimental setting, we analysed data from V1 of visually evoked activity in anesthetized macaque (*Macaca fascicularis*) monkeys. Resulting recordings were mostly confined to layers 2/3, an area where orientation and direction selectivity are cortical response properties prominently observed. The data were collected in the Laboratory of Adam Kohn at the Albert Einstein College of Medicine and downloaded from the CRCNS website [42]. Hence, the dataset is taken from previous work where the experimental procedures are described in detail [38–40]. Briefly, extracellular recordings were performed using Utah microelectrode arrays inserted 0.6 mm into cortex. Animals were paralyzed to minimize eye movements. All experimental procedures complied with guidelines approved by the Albert Einstein College of Medicine of Yeshiva University and New York University Animal Welfare Committees.

The spiking activity of neurons was recorded while presenting full-contrast drifting sinusoidal gratings presented at 12 orientations spaced equally (30°). Drifting gratings were presented binocularly for 1.28 seconds and separated by 1.5 seconds intervals during which a gray screen was presented. Stimulus orientation was randomized, and each stimulus was presented 200 times (i.e.,trials). The evoked dataset consisted of spiking activity from 59 to 105 neurons from 3 monkeys (dataset 1, 2, and 3, respectively). To characterize neuronal responses, we chose dataset 3, which included the most amount of neurons (105) out of all 3 datasets. For each orientation of the stimulus moving in the bidirectional motion, the trial-averaged firing rate of individual neurons was computed.

In V1, and other areas of the brain, neurons exhibit high trial-by-trial fluctuations in firing rate [43]. Regardless of the nature of the stimulus and the behavioural state of the animal, a widespread feature of cortical responses is the reduction in trial-by-trial variability around 100 ms following the onset of the stimulus [44]. Given that stimulus onset quenches neuronal variability, estimated neuronal responses following a certain delay would in turn provide a more accurate response representation of visual information. Hence, we computed the firing rate of individual cells during the remaining 1 second of the recordings, rather than the entire 1.28 seconds to ensure a decline in neuronal response variability. Furthermore, to remain consistent with the paradigm of the real-time robotic implementation, we analysed the spiking activity of 6 neurons in the dataset. While neuronal responses for all orientation gratings were analysed, we focused on finding neurons exhibiting higher responses exclusively for the vertical orientation gratings; the same orientation was processed by using the camera of the robot. Consequently, we chose 3 neurons (30, 63, and 103) from the dataset exhibiting preferential responses for vertical sinusoidal gratings moving towards the right and nonpreferential responses in the opposite null direction. Conversely, we chose 3 other neurons (5, 42, and 98) from the same dataset exhibiting preferential responses to stimuli moving left and nonpreferential responses in the opposite null direction.

## 3. Results

**Figure 5:**
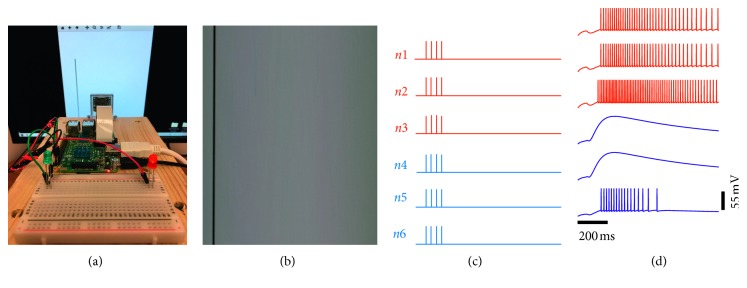
Right motion discrimination. (a) Activation of the red LED in response to a stimulus moving towards the right. (b) Real-world stimulus processed by using the Raspberry Pi camera. (c) Trains of presynaptic spikes fed to each unit in the microcircuit. (d) Postsynaptic response of individual units.

Figure 5 illustrates the visually evoked activity of individual units, where an orientation stimulus exhibits a rightward motion along a single axis. In this scenario, the activity of the microcircuit is dominated by the response of units within subpopulation 1, where units within this subpopulation exhibit a preferential response for stimuli moving towards the right. Higher responses for right motion discrimination are indicated by the activation of the red LED (Figure 5(a)).  Figure 5(b) illustrates a snapshot of the image captured by the camera during the time at which the stimulus is moving towards the right.  Figure 5(c) displays the presynaptic input to each unit within the microcircuit, whereas Figure 5(d) illustrates the visually evoked activity of individual units within the microcircuit during right motion discrimination.

**Figure 6:**
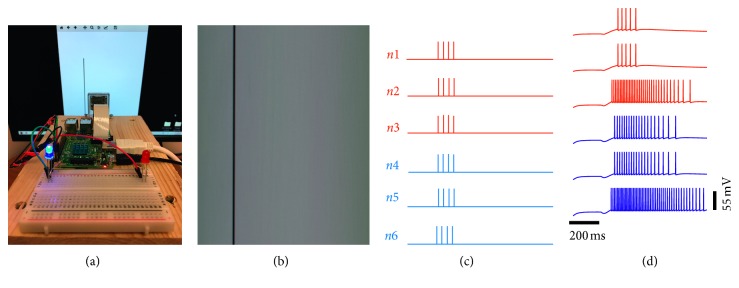
Left motion discrimination. (a) Activation of the blue LED in response to a stimulus moving towards the left. (b) Real-world stimulus processed by using the Raspberry Pi camera. (c) Trains of presynaptic spikes fed to each unit in the microcircuit. (d) Postsynaptic response of individual units.

Figure 6 illustrates the activity of individual units to a stimulus moving towards the left. Here, the activity of the microcircuit is dominated by the response of units within subpopulation 2. Higher responses from subpopulation 2 are in turn represented by the activation of the blue LED (Figure 6(a)). An image of the vertical orientation stimulus is displayed in Figure 6(b), resulting in direct incoming action potentials in the microcircuit illustrated in Figure 6(c).  Figure 6(d) illustrates the visually evoked activity of individual units within the microcircuit during left motion discrimination. A video illustration of the real-time robotic implementation and the corresponding spatiotemporal patterns of activity of all six units can be found in Supplementary Material (see S1 for video illustration).

**Figure 7:**
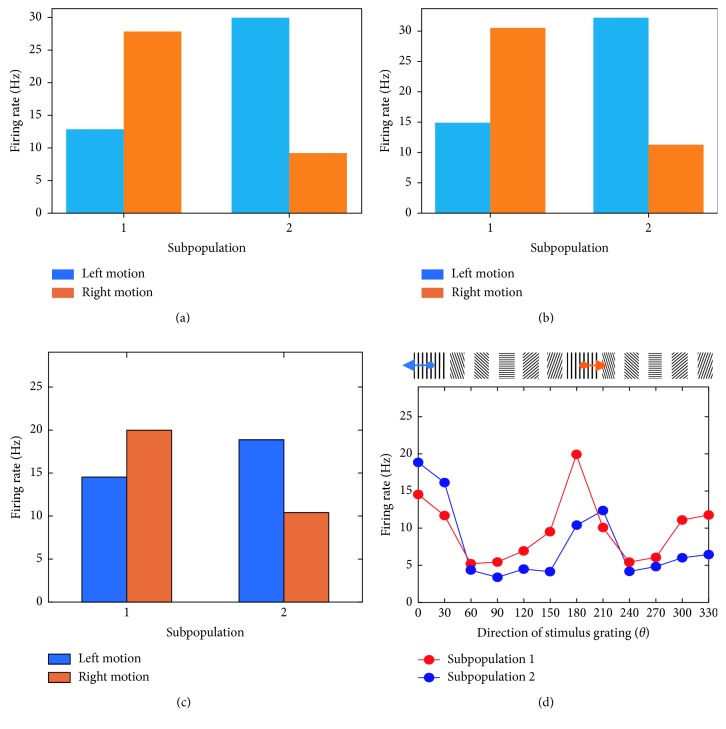
Response of 2 subpopulations during the simulation, real-time robotic implementation, and microelectrode recordings of V1. (a) Responses to hypothetical stimuli tested in the model. (b) Responses to real-world stimuli tested in the robot. (c) Responses to vertical sinusoidal gratings tested on a macaque monkey. (d) Responses to sinusoidal gratings presented at 12 orientations tested on a macaque monkey.

Figure 7 displays the average firing rate of units within subpopulations 1 and 2 exposed to stimuli moving in bidirectional motion. The visually evoked response of both subpopulations is shown when the model is exposed to hypothetical stimuli (Figure 7(a)) and real-world stimuli (Figure 7(b)). Units within subpopulation 1 exhibit a preferential response to orientation stimuli moving towards the right. Conversely, units within subpopulation 2 show a higher response to stimuli moving towards the left. Under both scenarios, the average firing rate of units in the preferred and nonpreferred direction is highly close to that observed amongst direction-selective neurons in V1 responding to drifting sinusoidal gratings (Figure 7(c)).  Figure 7(d) displays the average response of the direction-selective cells of interest across all orientations and directions. Among these responses, those resulting from vertical gratings are displayed in Figure 7(c). In subpopulation 1, the trial-averaged response of 3 cells shows preferential responses for right motion. In subpopulation 2, the trial-averaged response of 3 cells displays preference for left motion.

**Figure 8:**
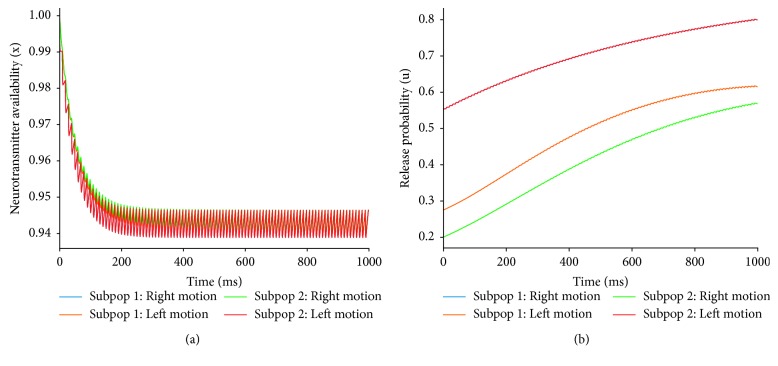
Parameters related to the efficacy of synaptic transmission during left and right motion discrimination. (a) Average synaptic resources available within each subpopulation. (b) Average neurotransmitter release probability in the synaptic connections within each subpopulation. Notice that the temporal evolution of the release probability is the same for subpop 1-right motion and subpop 2-left motion because their average initial release probability recruited for left and right motion is the same (Figure 4(b)).

Figure 8 illustrates the temporal dynamics of the synaptic variables *x*_*j*_(*t*) and *u*_*j*_(*t*) as the microcircuit process visual information. During motion discrimination, units within both subpopulations display synaptic connections that require similar amounts of synaptic resources available in order to properly mediate the response of both subpopulations. Furthermore, the average amount of neurotransmitters available is kept within a high range across the entire temporal domain (Figure 8(a)). This suggests that units within the microcircuit are minimizing use-dependent alterations of synaptic transmission during bidirectional motion discrimination, a scenario that is particularly advantageous when future task demands are required for the robot to perform. Finally, the examination of the release probability in the synaptic connections suggests that units within subpopulation 1 exhibit a higher neurotransmitter release probability for stimuli moving towards the right. On the contrary, synaptic connections within subpopulation 2 display a higher neurotransmitter release probability for stimuli moving towards the left (Figure 8(b)). This suggests that when the microcircuit is exposed to stimuli moving in a specific direction, units that exhibit preferential responses to stimuli moving in the specified direction are most likely to be mediated by synaptic connections that exhibit a high release probability [45].

**Figure 9:**
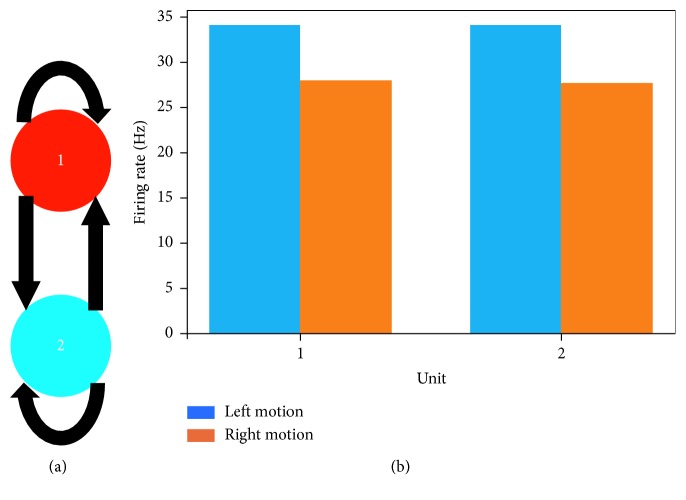
Direction selectivity in the two-unit model. (a) Architecture of the microcircuit. There are 2 units in total. (a) Outgoing connections from unit 1 and unit 2 exhibit STF. Connections are bidirectional, with self-connections allowed. (b) Average response of two units to a hypothetical stimulus moving in bidirectional motion. Unit 1 and unit 2 exhibit the same preference in response to opposite directions of motion. Besides keeping all other parameters the same, the default multiplicative factor *A* (Table 1) was multiplied by 2, in order to ensure that the mean activity of units falls within a similar range as that of the six-unit microcircuit (Figures 7(a) and 7(b)).

Next, we examined whether the topological structure of our expanded network adds functionality that would otherwise be absent in a two-unit microcircuit. In order to maintain a consistent comparison between the expanded network and the two-unit microcircuit, all of the synaptic connections were mediated by STF (Figure 9(a)). In addition, we examined the activity of each unit using the same kinetic parameters *τ*_f_ and *τ*_d_ (Table 1). Furthermore, we presented the same hypothetical stimulus in motion and recruited the same pairs of initial release probabilities *U* (Figure 4(a)). Under this framework, both units display highly synchronized spatiotemporal patterns of activity to stimuli moving in bidirectional motion (see S2 in Supplementary Material for video illustration). In addition, the two-unit microcircuit behaves as a single subpopulation, displaying preference only for a single direction of motion (Figure 9(b)). In contrast, the topological structure of the expanded network divides units into two distinct subpopulations, each of which displays preference for a direction opposite to that of its neighbouring subpopulation (Figure 7(a)). Taken together, the two-unit model limits the functional contribution of the microcircuit by displaying preference only for a single direction of motion, whereas the expanded architecture embodies a topological structure that lays the foundation for displaying preferential responses to both directions of motion.

**Figure 10:**
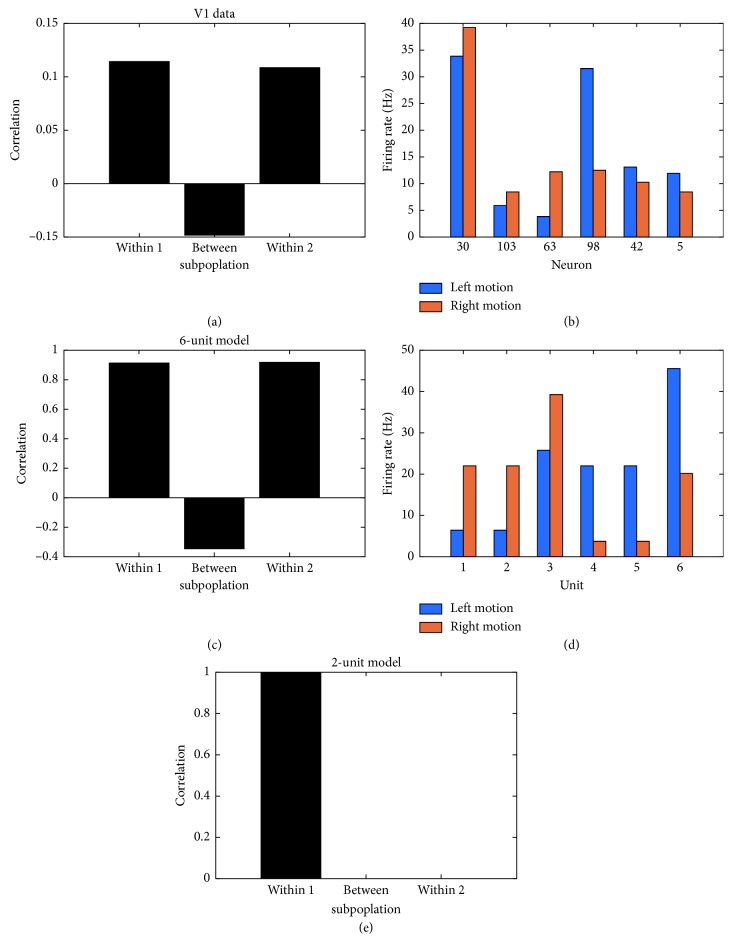
Spike count correlations predict shared and unshared motion direction preferences. (a) Mean SCC between pairs of V1 cells within and between subpopulations. (b) Average firing rate of six neurons in V1 responding to drifting vertical sinusoidal gratings. Neurons 30, 103, and 63 exhibit a preferential response for stimuli moving towards the right, whereas neurons 98, 42, and 5 exhibit a preference for stimuli moving towards the left. (c) Mean SCC between pairs of units within and between subpopulations in the six-unit microcircuit. (d) Average firing rate of six units responding to a vertical orientation stimulus in motion. Units 1, 2, and 3 exhibit a preferential response for stimuli moving towards the right, whereas units 4, 5, and 6 exhibit a preference for stimuli moving towards the left. (e) Mean SCC between the pair of units within the two-unit microcircuit. Given that both units display fully synchronized spatiotemporal patterns of activity for left and right motion discrimination (see S2 in Supplementary Material for video illustration), their mean SCC is 1. Notice that the two-unit model behaves as a single subpopulation; therefore, mean SCC for “Between” and “Within 2” cannot be computed.

In cortical microcircuits including V1, neurons exhibit shared fluctuations in population activity overtime [46]. In general, these shared fluctuations are measured between pairs of neurons over multiple presentations of an identical stimulus. To examine these coordinated fluctuations in spiking activity, we used a measure of spike count correlation (SCC) between pairs of neurons in V1 during motion discrimination [47]. In doing so, we used a nonoverlapping time window of 1 millisecond to compute the total number of spikes emitted from each neuron at a given time step. In this way, we obtained the total spike count of individual neurons across time over multiple presentations of the same stimulus in motion. We then computed the pairwise correlation coefficient matrix between 6 neurons, representing the SCC between all pairs of neurons. Finally, we computed the mean SCC observed between pairs of neurons within and between subpopulations. In doing so, we find a positive SCC within subpopulations, and a negative SCC between subpopulations (Figure 10(a)). Interestingly, a positive “within” SCC predicts that fluctuations in the activity of neurons within subpopulations are accurate predictors of a shared preference for a particular direction of motion (Figure 10(b)). Conversely, a negative “between” SCC predicts the presence of an unshared motion direction preference between units belonging to distinct subpopulations (Figure 10(b)). These results were qualitatively captured by our expanded microcircuit of six units (Figures 10(c) and 10(d)). In contrast, the microcircuit of two units displays preference only for a single direction (Figure 9(b)) and therefore fails to predict the presence of an unshared motion direction preference (Figure 10(e)). Taken together, the expanded architecture has a greater predictive power over the two-unit microcircuit, by exhibiting fluctuations in population activity that marks the presence of both shared and unshared motion direction preferences.

## 4. Discussion

Our simple and reproducible robotic implementation highlights the relation between short-term dynamics of synaptic transmission and motion discrimination. In our work, real-world stimuli are used and directly incorporated in a microcircuit dominated by a ubiquitous plasticity rule inspired from biological networks. As the microcircuit receives inputs, spiking units exhibit both preferential and nonpreferential responses to stimuli moving bidirectionally along a single axis of motion. Results from the simulation and the robotic implementation are in close agreement to analyses of visually evoked activity in V1, whereby cortical neurons exhibit higher responses for stimuli moving in the preferred direction of motion and lower responses for stimuli moving in the opposite null direction. In addition to accurate motion discrimination, the firing rate of motion-selective units in the STP model is close to the firing rate of direction-selective neurons in V1. As a result, the robotic implementation and the simulated version of the model capture both qualitative and quantitative depictions of typical neuronal responses observed in V1. In addition, units that exhibited preferential responses to stimuli moving in the specified direction were more likely to be mediated by synaptic connections exhibiting a high release probability. Furthermore, the contribution of STP as a complementary mechanism for direction selectivity is validated by the robotic implementation in real-time, showing successful motion discrimination at the behavioural level. By comparing neuronal responses from the robotic implementation to those of a simulated version of the model, accurate motion discrimination is observed despite the inherent noise of real-world stimuli present in the real-time robotic implementation. In addition, motion discrimination is conserved despite hardware constraints introducing differences in the timescale required to process stimuli in real-time (1398 seconds), versus the time required to run the computer simulation using hypothetical inputs (217 seconds). Here, processing time was measured using the execution time module named “*timeit*” in the *Python* programming language. In addition, the term “real-time” in our study referred to the amount of time it took for the Raspberry Pi microprocessor to run the STP model during which the camera processed 200 snapshots of the stimulus moving in bidirectional motion on the computer monitor. Hence, the term “real-time” was used in order to create a distinction between the robotic implementation and the simulated version of the STP model in a laptop computer.

Although visual experience exerts an influence over the direction preference that neurons acquire, the initial topological structure is an essential determinant of direction selectivity [48, 49]. In this work, the topological structure of the six-unit microcircuit expanded the repertoire of direction preferences over the two-unit microcircuit, allowing two subpopulations of units to exhibit preferential responses to opposing directions of motion. In contrast, this functional contribution happened to be absent in both the current and previously proposed [34] architecture of two units. In addition, although units in the two-unit microcircuit exhibited motion-induced progressive changes in their spatiotemporal patterns of activity, those spatiotemporal patterns were synchronized across both units during opposite directions of motion [34]. This functional property is in stark contrast to cortical networks, where asynchronous activity is more commonly observed across cells [50]. With the addition of more units, our larger network embodied a topological structure which inevitably added asynchronous spatiotemporal patterns of neural activity during motion discrimination. Finally, within the context of a larger network size, we show that global fluctuations in population activity can provide an accurate prediction of shared versus unshared motion direction preferences. More specifically, units within subpopulations displayed a positive SCC and were therefore more likely to exhibit fluctuations in subpopulation activity that were accurate predictors of a shared motion direction preference. In contrast, units between subpopulations shared a negative SCC, suggesting that units between subpopulations were likely to display preference for opposing directions of motion. This later prediction was absent in the two-unit microcircuit because the architecture behaved as a single microcircuit thus preventing neurons from displaying the presence of an unshared motion direction preference.

A predominant view from recent computational work suggests that direction biases present at eye opening may arise purely from “innate” network connectivity [6]. The onset of this architecture is suggested to be present in the absence of any explicit coding for direction selectivity and prior to any self-organizing process facilitated by spontaneous activity or motion-induced training [6]. Similarly, the topological structure of our expanded network was constructed in the absence of any explicit coding for direction selectivity. Hence, our work is in-line with recent experimental and computational studies suggesting that visual experience may serve a *permissive* role to complement structural processes that are fully characterized at the onset of visual experience [6, 48, 51, 52]. Hence, the organization of the initial architecture may lay the foundation for the map of direction preference, as observed in the visual cortex [49].

Visually evoked activity is likely to be mediated by a variety of mechanisms operating at different timescales and at distinct developmental stages [53]. Therefore, given the wide range of plasticity rules [54], it is likely to expect other candidate mechanisms that are complementary to short-term changes in synaptic strength. Indeed, there is experimental evidence within the literature largely supporting the interaction between STP and long-term synaptic plasticity [55–59]. Amongst long-term changes in synaptic strength, spike timing-dependent plasticity (STDP) has been proposed as a ubiquitous mechanism that strengthens or weakens synapses based on the relative timing of action potentials. Despite operating at different timescales, multiple experimental studies have shown that STP and STDP interact [11, 59–64]. As a potential application of real-time learning of visually evoked activity, a future extension of our work aims to combine STP with STDP. An examination of the synergistic interaction between STP and STDP would allow us to highlight the functional role of this interaction during visual information processing. This issue is of particular importance because it remains an open question as to how short-term changes in synaptic plasticity work their way in reorganizing a local neuronal circuit with STDP [54]. Given the importance of applying real-time learning in robotic systems, we intend to implement the extended model in a physical robot as a method for validating the functional role of the interaction between STP and STDP. This approach will allow us to examine the synergy between these two ubiquitous mechanisms, as learning unfolds in the developing circuit designed to perform motion discrimination of real-world stimuli from the sensory environment.

## 5. Conclusion

It is important to note that the work presented here does not provide a complete biophysical interpretation of the underlying neural computations observed in the brain. There are a variety of computational models in the literature that reside at different levels of description, with various levels of biological detail. Amongst these models, there are trade-offs of cognitive fidelity against biological fidelity [65]. The model proposed here presents itself as a model that exhibits motion discrimination, with a complementary mechanism that captures neuronal components designed in aiding the description of neuronal dynamics that transfer at the behavioural level. Hence, the phenomenological model is not designed to capture only cognitive function, nor is it designed to capture only neuronal components and dynamics. For example, synaptic transmission is a stochastic process whereby neurotransmitter release is unreliable. Hence, from a biophysical standpoint, an incoming action potential does not guarantee the triggering of neurotransmitter release. In contrast, the STP model proposed here captures the phenomenology of vesicular release. In doing so, the model assumes that the stochastic recovery of the vesicle is eliminated by either pooling the response from many independent synaptic connections or by taking a trial-averaged response of the stochastic recovery of the vesicle from a single synaptic connection. Hence, the model does not catch the full complexity of the nature of synaptic release. Despite the assumption of a deterministic model over a stochastic one, the phenomenological model has been formulated with strong support from electrophysiological data, capturing response traces accurately fitted by the *averaged* model [1]. Consequently, the phenomenological model of STP is detailed enough to support analyses of experimental data and general enough to transfer its applicability in a neurorobotic domain; capturing some aspects of cognition at the behavioural level, while staying grounded to fundamental biological processes. The proposed model therefore resides at a level of description that falls between the two ends of the spectrum, with a characterization of information processing that is useful when describing the performance of some task with some level of phenomenological abstraction. Consequently, the model proposed here turns the trade-off between full complexity and full cognitive function into a synergy between the two ends of the spectrum. At the theoretical level, the current approach in modelling neurobiological components intends to study neuronal dynamics and their contribution to cognition and behaviour. Hence, the phenomenological model is designed to be interpreted in the context of the cognitive functions it supports. Currently, cognitive models steer away from neurobiological fidelity, yet successfully implement task-performing cognitive models of the brain. These models take sensory inputs and exhibit motor outputs that perform experimental tasks that are well in-line with human-level performance. Conversely, biophysical models capture biologically plausible dynamical components of the brain with a high degree of fidelity but fail to exhibit cognitive task performance [65]. Hence, the neurobiological basis of the work presented here is intended to tie computational neuroscience to tasks of cognitive science, while being mindful of the compromise between biological plausibility and computational feasibility. As behaviour is deeply coupled not only in the underlying neuronal dynamics, but also by the anatomical constraints of the physical body it controls, the overall aim of this study was to provide a step forward in applying well-established models from neuroscience into the domain of neurorobotics. In doing so, it highlights the contribution of STP predominantly in the context of a motion discrimination task applied in a neurorobotic domain and uses an embodied robot as a method for qualitatively and quantitatively capturing the response characteristics of direction-selective cells in V1.

## Figures and Tables

**Table 1:** Short-term synaptic plasticity parameters.

Parameters	Values
	STF
Facilitation recovery (*τ*_f_)	750 ms
Depression recovery (*τ*_d_)	50 ms
Initial neurotransmitter availability (*x*)	1
Multiplicative factor (*A*)	0.039

**Table 2:** Leaky integrate-and-fire parameters.

Parameters	Values
Spike emission threshold (*θ*)	−55 mV
Resting membrane potential (*V*_rest_)	−70 mV
Membrane resistance (*R*_m_)	200 mΩ
Membrane time constant (*τ*_m_)	30 ms
Absolute refractory period (*τ*_arp_)	2 ms
Integration time step (*dt*)	1 ms
Stimulus duration (*T*)	1000 ms

## Data Availability

The experimental data used to support the findings of this study were supplied by Adam Kohn under license and so cannot be made freely available. Requests for access to these data should be made to Jeff Teeters, jteeters@berkeley.edu.

## Supplementary Materials

Supplementary Materials S1. A video clip of the real-time robotic simulation setup and the corresponding spatiotemporal patterns of activity of all six units. S2. A video clip of the spatiotemporal patterns of activity of the two-unit model.
